# Carbon Dot Nanoparticles: Exploring the Potential Use for Gene Delivery in Ophthalmic Diseases

**DOI:** 10.3390/nano11040935

**Published:** 2021-04-06

**Authors:** Manas R. Biswal, Sofia Bhatia

**Affiliations:** 1 Department of Pharmaceutical Sciences, Taneja College of Pharmacy, University of South Florida, Tampa, FL 33612, USA; bhatias@usf.edu; 2 Department of Internal Medicine, Morsani College of Medicine, University of South Florida, Tampa, FL 33612, USA; 3 Department of Ophthalmology, Morsani College of Medicine, University of South Florida, Tampa, FL 33612, USA; 4 Department of Molecular Genetics and Microbiology, College of Medicine, University of Florida, Gainesville, FL 32610, USA

**Keywords:** photoluminescence, biocompatibility, clathrin- and caveolae-mediated, ocular gene delivery

## Abstract

Ocular gene therapy offers significant potential for preventing retinal dystrophy in patients with inherited retinal dystrophies (IRD). Adeno-associated virus (AAV) based gene transfer is the most common and successful gene delivery approach to the eye. These days, many studies are using non-viral nanoparticles (NPs) as an alternative therapeutic option because of their unique properties and biocompatibility. Here, we discuss the potential of carbon dots (CDs), a new type of nanocarrier for gene delivery to the retinal cells. The unique physicochemical properties of CDs (such as optical, electronic, and catalytic) make them suitable for biosensing, imaging, drug, and gene delivery applications. Efficient gene delivery to the retinal cells using CDs depends on various factors, such as photoluminescence, quantum yield, biocompatibility, size, and shape. In this review, we focused on different approaches used to synthesize CDs, classify CDs, various pathways for the intake of gene-loaded carbon nanoparticles inside the cell, and multiple studies that worked on transferring nucleic acid in the eye using CDs.

## 1. Introduction

Over the last decade, gene therapy has presented as a possible and promising treatment for various genetic disorders, such as Parkinson’s disease and immunodeficiency diseases, and has attracted significant attention in the field of medical sciences. The straightforward principle behind gene therapy is to correct the disease origin by delivering missing or defective gene products [1]. However, therapeutic approaches vary depending on the type of gene and mutation. Luxturna, the first Food and Drug Administration (FDA)-approved ocular gene therapeutic product was developed to treat Leber congenital amaurosis (LCA) patients with bi-allelic mutations in the RPE65 gene. Most of the ongoing therapies in different clinical trial phases are based on adeno-associated virus (AAV) vectors [2]. Due to small size capacity, high production costs, high immunogenicity, inflammatory responses, and invasive administration to the retina, viral gene delivery raises concerns [3]. This brings up basic research on non-viral vectors, such as different kind of inorganic nanoparticles (gold nanoparticles [4], magnetic nanoparticles [5], carbon nanotubes [6], nanodiamonds [7], graphene [8]) for nucleic acid delivery [9]. Moreover, NPs overcome various safety concerns as compared to viral vectors, such as cost-effectiveness, ease to customize, design, and optimize for cellular uptake, large DNA capacity which is a very critical factor to deliver large ocular disease gene.

The combination of nanotechnology and medicine led to the emergence of nanomedicine whereby a nanomaterial platform is used for gene or drug delivery with diagnostic probe [10,11]. The enhances permeability and retention (EPR) effect has been a crucial basis for the development of Nano medicines [12]. Due to the EPR effect, the therapeutic effect of drug-loaded nanoparticles with appropriate size increases significantly [13]. In a study, Ideta et al. [14] confirmed the delivery of a poly ion complex (PIC) micelle encapsulating fluorescein isothiocyanate-labeled poly-L-lysine (FITC-P(Lys)) targeted to the choroidal neovascularization (CNV) lesions in rats. The results showed that PIC micelles had long-circulating features and were retained in highly permeable CNV lesions for 168 h after intravenous administration. These micelles were probably accumulating in CNV lesions through the EPR effect. These days, nanomedicine is a rapidly growing research area that aims to apply nanoparticles like metallic nanoparticles, semi-conductive quantum dots, carbon materials like graphene oxide or nanotubes for therapeutics, imaging, sensing, and stimuli-responsive carriers. Out of these, carbon dots have been identified as potential material for various nanomedicine applications [15,16]. Carbon-based nanoparticles are made only of carbonaceous nanomaterials. CDs were first discovered and observed by Xu et al. [17] during the separation of single-walled carbon nanotubes from carbon soot using the arc discharge method. C-dots are spherical shape nanocarriers with a diameter of <10 nm [18]. They possess a carbon core with different functional groups (e.g., -OH, -COOH, -NH_2_) on their surface which provides high biological activity, good water solubility ability to form conjugates with other organic and inorganic substances [19]). Due to the properties like extremely small size, ease of synthesis and modification, low cytotoxicity, the higher degree of oxidation, and good water retention [20], CQDs have been used in a wide number of the application; Fluorescent labeling and cell imaging [21], targeted drug delivery for cancer treatment [22], detecting heavy metal ions [23], bacterial labeling and detecting live/dead bacterial differentiation using charge-selective interaction [24].

**Figure 1:**
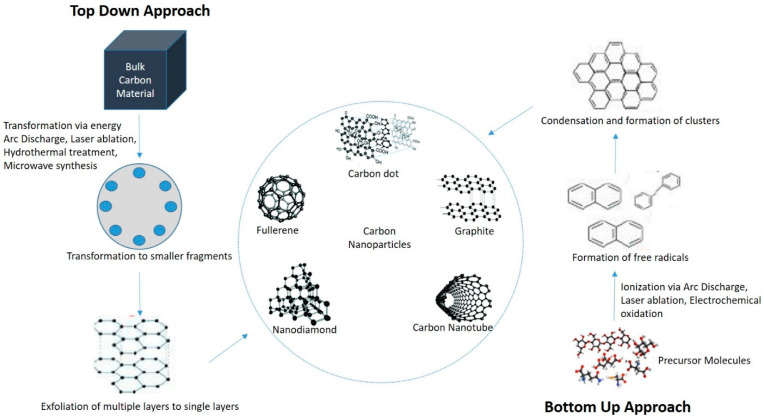
Various approaches and methods used to form carbon-based nanoparticles.

The CDs synthesis procedures can be divided into two groups, namely top-down and bottom-up methods [25] (Figure 1). Various top-down synthesis methods such as laser ablation, arc-discharge, hydrothermal and electrochemical oxidation involve the breaking of bulk carbon precursors into nanosized particles [26]. The bottom-up method involves dehydration, polymerization, and carbonization of small carbon molecules which result in the formation of graphene quantum dots, and amorphous carbon nanodots via hydrothermal synthesis, microwave pyrolysis and solution chemistry [27]. Table 1 shows different fabrication methods used for CDs synthesis and their advantages and disadvantages.

There are different types of CDs such as graphene quantum dots (GQDs), carbon quantum dots (CQDs), carbon nanodots (CNDs), carbonized polymer dots (CPDs) depending upon the structure of carbon core, surface groups, and properties. GQDs consist of single or few layers of graphene sheets. GQDs have known to have better crystalline structure relative to CDs as the presence of sp^2^ carbon units in CDs have lower crystallinity as compared to GQDs [34]. CQDs constitute multilayered graphite structures connected with surface groups. CNDs are highly carbonized nanoparticles without obvious crystallinity. CPDs possess a polymer/carbon hybrid structure including several functional group chains on the surface and a carbon core [35].

## 2. Properties of CDs

CDs have attracted researcher because of their unique optical and physicochemical properties [36] which are discussed in this section.

### 2.1. Absorption

CDs showed strong absorbance in the 280–360 nm UV region with tail extending into visible region where absorption band are assigned to n–π * transition of C=O/C=N bond or π–π * transition of C=C bond [37,38]. The absorption band and emission properties of CDs can be regulated by types and content of surface modifications or surface functional groups, π-conjugated domains or variation of oxygen/nitrogen content in carbon core.

### 2.2. Photoluminescence

Photoluminescence (PL) is one of the most significant properties of CDs which make it an important material for biomedical applications. The PL nature of CDs depends on the size, morphology, concentration, internal structure, and composition of the particle. These features are regulated by the initial precursors and fabrication methods used to form CDs. Various methods such as hydrothermal carbonization and microwave synthesis can be used to elevate the PL of CDs by surface alteration. Various studies have reported that surface passivation could increase the PL activity of CDs. Some of the commonly used surface passivating agents are polyethylene glycol (PEG) and polyethyleneimine (PEI). Heteroatom-doped CDs have also been used to adjust the intrinsic features. Sahiner et al. [39], reported an easy and fast method to prepare N- and S-doped amino acid derived CDs in a single step using microwave irradiation in 2 min which increases the photoluminescence efficiencies.

### 2.3. Quantum Yield (QY)

QY of CDs is also an important parameter that depends on the raw material and fabrication method. It has been found that CDs fabricated from graphite, citric acid, and candle soot reported QY up to 10%. Various studies have reported various approaches such as surface passivation or modification and element doping with nitrogen and sulfur [40] to increase the QY of CDs. Xu et al. [41] prepared sulfur-doped CDs with 67% QY using the hydrothermal method. A study done by Zhuo et al. [42] reported 80% of QY while fabricating CDs using citric acid and glutathione as starting material. Hu et al. [43] prepared polyethyleneimine-based carbon dots (PCD) by the oxidation of polyethyleneimine with a QY of 54.3%.

### 2.4. Biocompatibility

It is very complicated to determine the cytotoxicity of carbon nanoparticles. Various factors such as the presence of metal catalysts/graphite, surface coating, UV radiation exposure, dispersion properties, aggregation due to high Van der Waals forces affect the behavior of these particles [44]. Many studies investigated the cytotoxicity of CDs in vitro [11,45,46] as well as in vivo. In 1999, Huczko et al. [47] studied the toxic effects of fullerene matter on Draize rabbit and found no effects on the eye within 24, 48, and 72 h, respectively. Later, Aoshima et al. [48] performed a toxicity study to find the safety of fullerenes used in the cosmetic industry on rabbit eyes. They found that fullerenes could cause redness in the conjunctiva and defects in corneal epithelia, but the symptoms disappeared in two days. However, it is difficult to know its effect on humans as most of the research was done on rat and rabbit eyes which are more sensitive to particles. Some of the latest studies reported non-toxicity in vitro experiments in CDs [32,49]. Zhang et al. [50] incubated CT26.WT and CAL-26 cells with 0.1–1 mg/mL CDs solution and evaluated the cytotoxicity of CDs on cells using MTT assay. The viability of cells after 24 h was found to be >84% for CT26.WT cells and >60% for CAL-27 cells at the concentration of 1 mg/mL. In another study, Li et al. [51] evaluated cytotoxicity on fabricated C-dot-based nanohybrids with multihydroxy hyperbranched polyglycerol (HPG) at the concentrations of 0.1–10 mg/mL. The study reported ≥90% cell viability at the concentration of 0.1–2.0 mg/mL and it decreases at higher concentrations. However, it is critical to investigate the safety and toxicity profiles of nanomedicine formulations in order to stimulate clinical translations of nanomedicine [52]. The generation of reactive oxygen species (ROS) by nanoparticles may cause toxicity. ROS may cause potential adverse effects such as cellular components damage. Tabish et al. [53] reported association of ROS with graphene cellular toxicity. Graphene-induced toxicity depends on its physiochemical interactions with other organs where it might be accumulated. Graphene-induced ROS may cause oxidative stress, loss of cellular functions, mitochondrial damage, lipid peroxidation, nucleic acid modifications, and inflammation, which lead to cell death and toxicity. To date, the mechanism and roles of ROS produced by graphene have not been fully explored. A study done by Tabish et al. [54], demonstrated a facile approach to manufacture GQD with high single oxygen yield and good biocompatibility by assessing the toxicity of GQDs in vivo as well as in vitro.

It is difficult to synthesize uniform size CDs. The size distribution is important as it decides the toxicity and fluorescence nature of CDs and it may obstruct their biological applications [55]. Different synthesis methods may result in different size, yield, fluorescence color, which can obstruct CDs commercialization [55]. There are multiple studies on the cytotoxicity of CDs in different concentrations in various cell lines. However, the role of the synthesis approach on the toxicity of fabricated CDs has not been fully explored yet [56]. A study done by Esfandiari et al. [56] studied the effect of carbonization degree in the synthesis of four different CDs on cytotoxicity, photo-induced toxicity, and cellular uptake under different experimental conditions. The authors synthesized CDs via citric acid thermal decomposition at different temperatures and time durations. The size, QY, the toxicity of synthesized CDs was characterized. The study demonstrated that the small changes in the synthesis conditions may have significant effects on the properties of CDs.

## 3. How the CDs Deliver DNA/RNA to the Cells

**Figure 2:**
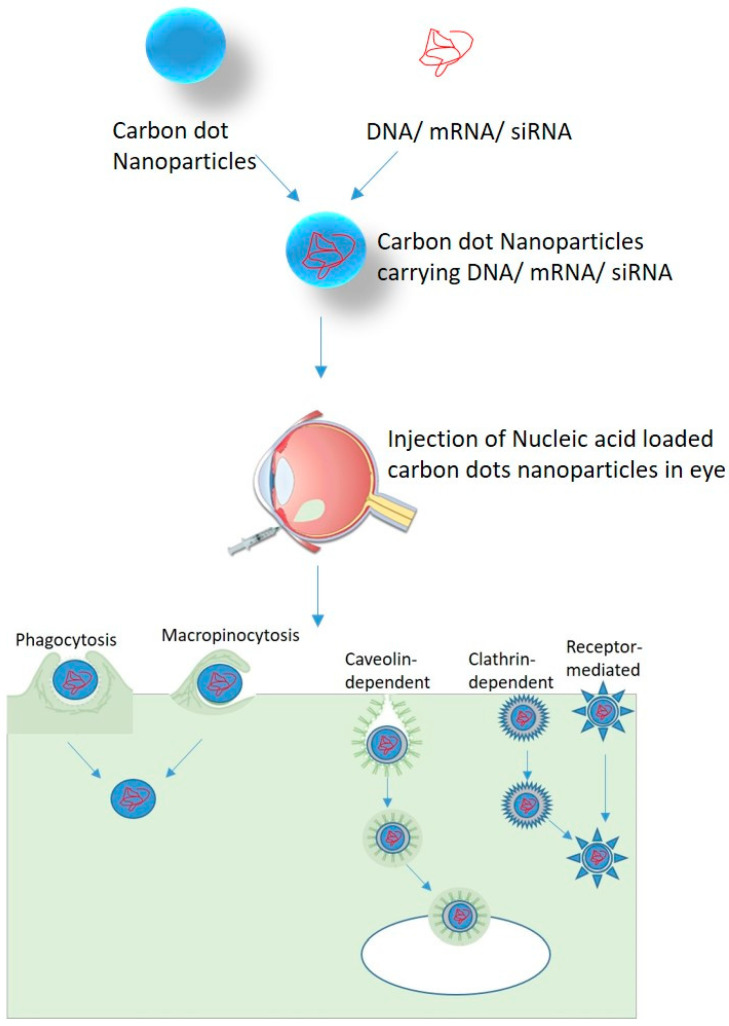
Nucleic acid delivery in retinal cells using carbon dots nanoparticles.

Carbon Nanoparticles can enter the cell by several processes such as phagocytosis [57], endocytosis, and micropinocytosis [58] (Figure 2). Endocytosis pathways can be classified into clathrin- and caveolae-mediated endocytosis, phagocytosis, macropinocytosis, and pinocytosis. The phagocytosis pathway is actin-dependent and limited to macrophages, dendritic cells, and neutrophils. They engulf foreign materials of large size (>0.5 um) [59]. Macropinocytosis is an actin-regulated process in which cells internalize fluids and particles together and form large size vesicles (0.2–5 um) [59]. Actin assembly plays an important role in the uptake process because of the micrometer length scale of phagocytosis and macropinocytosis [60]. Pinocytosis involves the absorption of extracellular fluids, small molecules and, forms small vesicles of 100 nm size [59]. Receptor-mediated endocytosis (RME) is known to be a potential method for NPs uptake and it includes clathrin- and caveolae-mediated endocytosis. These pathways are important for the cellular internalization of NPs. In clathrin-mediated endocytosis, receptor–ligand binding initiates the formation of coated pits on the cytosolic side of the plasma membrane which further forms a closed polygonal cage [61]. In caveolin-mediated endocytosis, the assembly of hairpin-like caveolin coats was formed on the cytosolic side of the plasma membrane [62]. Both clathrin- and caveolin-mediated pathways were reported to have highly complex biochemical cascades [63]. Various features, such as shape, size, and physicochemical properties, of NPs regulate the rate and quantity of cellular uptake of NPs. Carbon dots incubated with different mammalian cell lines showed penetration of a small amount of carbon into the cell membrane and also showed favorable biocompatibility. Four cellular uptake inhibitors (glucose, filipin III, 5-(N,N-dimethyl)-amiloride (DMA) and chlorpromazine hydrochloride (CPZ)) were used to study the internalization mechanism of CDs/pDNA nanomaterials. The results showed strong fluorescence emission by DMA and no fluorescence emission by filipin III, glucose, and CPZ. It has been observed that CDs could internalize both plasmid DNA and siRNA via clathrin- as well as caveolae-mediated endocytosis to achieve effective gene expression as compared to macropinocytosis [15].

## 4. CDs Efficiency in Gene Delivery

In recent years, CDs have been successfully used for in vivo as well as in vitro bioimaging, and gene therapy. Folic acid (FA), hyaluronic acid (HA), RGD-peptide have been used as fluorescent probes in conjugation with CDs for disease diagnosis and therapy. Properties such as biocompatibility, less cost fabrication technique, lower cytotoxicity, water-solubility, effective binding with organic and inorganic molecules, different intake routes make CDs better for gene delivery as compared to other non-viral vectors. PEI has been commonly used cationic polymers in gene delivery. Liu and coworkers, [11] constructed highly efficient dual-functional nanoparticles which are based on PEI-passivated carbon dots (C-dots) by microwave assisted pyrolysis of glycerol in the presence of branched PE125k in one-step and demonstrated the use of CDs for in vitro gene delivery. In this hybrid C-dot, PEI played two important roles: one is surface passivation to endow the C-dots with strong photoluminescence, and second, DNA condensation for gene transfection. The capability of DNA condensation and cytotoxicity of CD-PEI depends on the pyrolysis time. Authors found that the CD-PEI which are obtained at appropriate pyrolysis time showed lower toxicity, higher gene expression of plasmid DNA in COS-7 cells and HepG2 cells. The fluorescent emission at various wavelength showed internalization of CD-PEIs into the cells which make CD-PEI as a potential application in gene delivery and bioimaging. Wang et al. [32], synthesized a novel photonic carbon dots (Cdots) based nanocarrier using low molecular weight amphiphilic PEI (Alkyl-PEI2k) for surface passivation. The results showed that Alkyl-PEI2k-Cdot nanocarrier is water-dispersible and possesses good stability, fluorescence, monodispersity with a narrow size distribution, low toxicity in cells, and high gene delivery efficiency.

Wang et al. [64], experimented to construct carbon dots where hyaluronic acid (HA) was used as a carbon source and polyethyleneimine (PEI) as a passivating agent using the synthetic method with no additives and validated the use of CDs for in vivo gene delivery. FT-IR and NMR results showed that some residues of HA and PEI remain in the HA-CDs structure. It has been observed that these materials had lower cytotoxicity than PEI and good serum tolerance. The authors found higher transfection efficiency (up to 50 times) in the presence of 10% serum. HA-CDs showed good intracellular imaging ability and fluorescence emission was observed under various wavelengths.

He et al. [65], prepared two cationic polymer-derived C-dots (Taea-CD and Cyclen-CD) using the hydrothermal method which has the potential to condense DNA into nanoparticles with a positive charge and provide protection to DNA from degradation. The results showed 2000 times higher transfection efficiency and also showed high serum tolerance and cell viability as compared to commercially available PEI. Due to the optical property of C-dots, better efficiency in the transfection process was observed by cell imaging and real-time detection using confocal laser scanning microscopy (CLSM) assay. Another study was done by Chen et al. [9], prepared CDs from low molecular weight PEI and modified them with various hydrophobic chains and different degrees of substitution. Analytical approaches like ^1^H NMR spectroscopy, FT-IR spectroscopy, TEM, and XPS were used to confirm modification and substitution. Authors demonstrated the capability of CDs in bioimaging, and gene delivery and also found that the prepared CDs, Ole1.5-CD showed 200 times higher transfection than PEI 25 kDa in the presence of serum in A459 cells. Table 2 shows previously reported studies for the delivery of gene using CDs.

## 5. Applications of CDs in Ocular Treatments

Because of the unique properties of CDs, many studies have reported in vivo and in vitro gene delivery for different diseases using CDs. In recent times, some of the studies have reported the application of CDs in ocular gene delivery. A study done by Jian et al. [73] has synthesized carbon quantum dots (CQDs) from biogenic polyamines (PAs) and can be used as a promising antibacterial agent for the treatment of keratitis, a disease caused by microbial infections. High yield CQDs were synthesized using a one-step method by direct pyrolysis of spermidine (Spd) powder through a simple dry heating treatment. Authors demonstrated that CQDs obtained from Spd (CQD_Spds_) have effective antibacterial activities against multidrug-resistant bacteria (methicillin-resistant S. aureus) and non-multidrug-resistant bacteria (*E. coli*, *S. aureus*, *P. aeruginosa*, and *S. enterica serovar enteridis*). Higher minimal inhibitory concentration (MIC) indicates good antibacterial capabilities. Evaluation obtained from in vitro cytotoxicity, hemolysis, hemagglutination, genotoxicity, and oxidative stress and in vivo morphological and physiological corneal changes indicates better biocompatibility of CQD_Spds_. Hahn et al. [74] performed an experiment to treat retinitis pigmentosa (RP) using photosensitized-bonded carbon nanomaterial was covalently bonded to hyaluronic acid under lightless condition. The hyaluronic acid-carbon nanomaterial-photosensitizer complex prevents the formation of active oxygen in RPE cells for long period of time. The study manufactured CQD-Ce6 complex and HA-CQD-Ce6 complex and analyzed their effect on the prevention of active oxygen. It was confirmed that HA-CQD-Ce6 complex showed 80% oxygen suppression ability while CQD-Ce6 showed 60% in the cells.

Using an MTT assay, it was confirmed that HA-CQD-Ce6 complex does not show cytotoxicity until the concentration of Ce6 reaches 100 ng/mL. The RPE cells treated with HA-CQD-Ce6 and CQD-Ce6 was further treated with sodium iodate to check he cell viability. The results showed that the viability of cells in both complex treated RPE cells was lower as compared to sodium iodate treated cells. Authors also confirmed that RPE cells has hyaluronic acid receptor and they exhibit high cell uptake. The complex showed excellent therapeutic efficiency and very easily infiltrate in the cells.

Hasanzedah et al. [75] developed CD-PEI as a traceable multicolor photoluminescent gene delivery nanocarrier by pyrolysis of a mixture of citric acid and branched PEI using a microwave approach. This is the first study that reported the transfer of pCRISPR to the cells by CD-PEI with high quantum yield. PEI not only play role in condensing DNA on the surface of CDs but also help in passivation. In vitro study using HEK 293 cell line showed low cytotoxicity and acceptable transfection efficiency. Nanodiamond (ND), another carbon-based nanomaterial used to carry biomolecules such as DNA, protein, and small drug molecules. Yang et al. [76] tested the possibility of using Nanodiamonds (NDs) based CRISPR-Cas 9 delivery system as a tool for creating in vitro and in vivo disease models of X-linked retinoschisis (XLRS). Authors designed NDs-based CRISPR-Cas9 delivery vector by functionalizing carboxyl (-COOH) group to the NDs surface and covalently conjugated with fluorescent 6His-tagged mCherry reporter protein. mCherry protein is further conjugated with two linear DNA constructs; one Cas9 endonuclease with GFP reporter protein and another sgRNA containing HDR template insert which is designed to insert mutation (c.625C > T) in RS1, a gene associated with X-linked retinoschisis (XLRS) phenotype. These NPs were internalized by human iPSCs and mouse retinas via the endosome pathway. Authors demonstrated that mCherry protein remains stables in the retina for up to 2 weeks and the addition of BSA increases the efficiency of NDs.

Shoval et al. [77] performed a study to deliver an anti-VEGF aptamer across the corneal structure into the eye using a C-dots-aptamer hybrid system which inhibits the angiogenesis induced by VEGF in the posterior eye chamber. The hydrophobic properties of C-dots and hydrophilic properties of aptamer-functionalized surface domains increase the permeation of carriers through a highly complex corneal structure, consisting of hydrophilic stroma and hydrophobic epithelium. The fluorescence of anti-VEGF aptamer-functionalized C-dots helps in monitoring the intraocular concentration of drugs in the eye.

## 6. Conclusions

Many studies have made progress in utilizing various properties of CDs in optical, energy, and biomedical applications. A lot of research development is going on in CDs on their synthesis, structures, properties, and application development. In all carbon-based materials, research on CDs is still in a more emerging stage. So, there is a potential interest in producing good-quality CDs with desirable shape, size, crystallinity, number of functional groups, and types. In this review, we have briefly summarized types, synthesis methods, properties, different internalization pathways, and recent advances in the use of CDs in retinal gene therapy. Unique physicochemical properties such as photoluminescence and in vitro and in vivo studies showing the non-toxicity of CDs have also been discussed. The stable photoluminescence and low cytotoxicity of CDs make them a potential candidate for bio-sensors, bio-imaging, drug delivery, and gene/plasmid/RNA delivery. Surface passivation or modifications of CDs with chemical groups are beneficial for functional hybrids, fluorescent nanocomposites, and high refractive index materials [78]. The synthesis process and biological applications of CDs are believed to be safe and biologically benign. Due to this, CDs could become a promising candidate to replace organic and inorganic quantum dots in the future.

## Figures and Tables

**Table 1:** Advantages and disadvantages of different methods of C-dots synthesis.

Strategies	Fabrication Method	Carbon Source	Size (nm)	Yield (%)	Luminescence Wavelength (nm)	Advantages	Disadvantages	Ref.
Bottom-up	Thermal decomposition	sucrose	1.84	21.6	365	Less time consuming, easy to operate, low cost, large scale production	Broad size distribution	[28]
	Hydrothermal treatment	citric acid	2.69, 3.10	71, 78	420–520	Cheap, eco-friendly, lack of toxicity, low cost	Low yield	[29]
	Microwave synthesis	glucose	2.75, 3.65	6.3, 3.1	330	Fast, low cost, eco friendly	Poor size control	[30]
Top-down	Electrochemical/chemical oxidation	acetonitrile	2.8	6.4	365	High yield, high purity, low cost, control over size	Few small molecule precursors	[31]
	Arc discharge	Arc soot	18.0	-	365	Fabricate carbon NPs in a variety of gases	Require more purification	[17]
	Laser ablation					Easy control over size and photolumicense property	High cost and sophisticated process	[32]
	Ultrasonic treatment	Waste food	4.6	2.85	>400	Convenient to break large carbon materials, well dispersed, low crystallinity	High energy cost	[33]

**Table 2:** Studies reported use of CDs in gene delivery.

CDs	Precursors	Synthesis Method	Size (nm)	QY (%)	Ccell Lines	Findings	Ref.
CD-PEI	glycerol	Microwave pyrolysis	7–12	-	COS-7; HepG2	Constructed high efficient nano gene vector with strong photoluminence and efficient transfection	[11]
PCD	PEI	Hydrothermal reaction	3–4	54.3	MCF-7; 293T	Evaluated the use of PCD as fluorescence probe for cell imaging	[43]
CD-PDMA-PMPD	citric acid	Microwave	50	41.5	COS-7	Displayed higher transfection efficiencies and provide a promising platform for serum-resistant gene delivery and imaging	[66]
siRNA- Cdots@PEI	CA, tryptophan, Nitrogen	Microwave prolysis	4.7 ± 0.8	24.2	MGC-803	Demonstrated the intake of siRNA into gastric cancer cells MGC-803 causes gene silencing	[32]
CD/siRNA	Citric acid, branched PEI	Microwave prolysis	12–13.2	31.5–48.1	A549	Reported the potential of CDs/siRNA delivery in A549 cells for the treatment of lung disease	[67]
fc-rPEI-Cdots	Glycerol and PEI	Microwave pyrolysis	143.1	-	H460; 3T3	Showed potential in lung cancer targeting and treatment	[68]
Positive charge CDs	PEI and FA	Hydrothermal reaction	-	42	293T; HeLa	Reported a low cost synthesis method that exhibit photoluminescent property for cancer diagnosis and gene therapy	[69]
HP-CDs	branched PEI	Hydrothermal	2.25	12.4	HeLa	Evaluated great potential of HP-CDs in tumor targeting, intracellular imaging and gene delivery	[70]
CDs/pDNA	Porphyra polysaccharide-EDA	hydrothermal	<10	56.3	EMSCs	Demonstrated more efficient neuronal fifferentiation of EMSCs with CDs/pDNA	[71]
FCDs	Tetrafluoroterephthalic acid and branched PEI	Solvothermal process	136 ± 5	-	HEKT292	FCDs showed higher transfection even in high serum concentration and low DNA dose	[72]
CDs/pSOX9	Arginine, glucose	Microwave prolysis	10–30	12.7	MEFs	Successfully delivered pSOX9 into embryonic fibroblast cells of mouse and in vito results showed high gene transfection efficiency	[1]
